# A systematic review of clinical trials of pharmacological interventions for acute ischaemic stroke (1955-2008) that were completed, but not published in full

**DOI:** 10.1186/1745-6215-11-43

**Published:** 2010-04-22

**Authors:** Lorna M Gibson, Miriam Brazzelli, Brenda M Thomas, Peter AG Sandercock

**Affiliations:** 1 College of Medicine and Veterinary Medicine, University of Edinburgh, UK; 2 Department of Clinical Neurosciences, University of Edinburgh, Western General Hospital, Edinburgh, UK

## Abstract

**Background:**

We assessed the prevalence, and potential impact of, trials of pharmacological agents for acute stroke that were completed but not published in full. Failure to publish trial data is to be deprecated as it sets aside the altruism of participants' consent to be exposed to the risks of experimental interventions, potentially biases the assessment of the effects of therapies, and may lead to premature discontinuation of research into promising treatments.

**Methods:**

We searched the Cochrane Stroke Group's Specialised Register of Trials in June 2008 for completed trials of pharmacological interventions for acute ischaemic stroke, and searched MEDLINE and EMBASE (January 2007 - March 2009) for references to recent full publications. We assessed trial completion status from trial reports, online trials registers and correspondence with experts.

**Results:**

We identified 940 trials. Of these, 125 (19.6%, 95% confidence interval 16.5-22.6) were completed but not published in full by the point prevalence date. They included 16,058 participants (16 trials had over 300 participants each) and tested 89 different interventions. Twenty-two trials with a total of 4,251 participants reported the number of deaths. In these trials, 636/4251 (15.0%) died.

**Conclusions:**

Our data suggest that, at the point prevalence date, a substantial body of evidence that was of relevance both to clinical practice in acute stroke and future research in the field was not published in full. Over 16,000 patients had given informed consent and were exposed to the risks of therapy. Responsibility for non-publication lies with investigators, but pharmaceutical companies, research ethics committees, journals and governments can all encourage the timely publication of trial data.

## Background

Completing but not publishing clinical trials in full can be unethical[1]. Well designed clinical trials should be published because their results can benefit patients, justifying the risk to trial participants from experimental treatments[1]. Failing to publish clinical trials can bias the findings of systematic reviews and meta-analyses if their findings differ from published results[2] which may lead to inappropriate treatment decisions and health care policies[3].

Previous studies of unpublished clinical trials used small samples of clinical trials to estimate the prevalence of non-publication, including trial abstracts which were submitted to one conference over three years [4-6], trials submitted to a single research ethics committee[7,8] or a single country's drug regulatory agency[9]. Limiting searches for published trials to databases[4,8] may have missed full trial reports in non-indexed journals and books, leading such trials to be misclassified as unpublished. Also ongoing trials may have been misclassified as unpublished by failing to determine the status of clinical trials[4,6,9]. A systematic review which identified trials of acute ischaemic stroke only through reference lists of published Cochrane Stroke Group reviews found that 11% (19/178) had not been published in full as of December 31, 1999, although the trials had been reported in abstracts between two and 17 years before this date[10].

The Cochrane Stroke Group's Specialised Register of Trials is a very comprehensive database[11] which includes over 12,000 reports relating to around 6,000 stroke trials (Brenda Thomas, personal communication, June 2008). We used this register to perform a systematic review of clinical trials of pharmacological interventions for acute ischaemic stroke. We aimed to determine the prevalence of and characteristics of completed but unpublished trials.

## Methods

### Definition of 'unpublished'

We classified trials as unpublished if detailed methods and results were not reported in either a peer-reviewed journal article [8] or book chapter, i.e. not published in full. We considered forms of written information including abstracts, drug company reports, letters, literature reviews, and online trials registers to be less than full publications, which we term 'sources.' If it was unclear whether a trial had or had not been published from the titles of references, the trial source(s) were retrieved. We considered a study in the trials register to be unpublished if we could not match the details of study methods and results to any subsequent full publication.

### Search strategy

One author (BT) searched the Cochrane Stroke Group's Specialised Register of Trials[12] on June 19^th ^2008 for trials of pharmacological interventions for acute ischaemic stroke. Another author (LG) screened the titles of references identified from the search of the register to select trials which appeared to be unpublished. We searched MEDLINE and EMBASE from January 2007 to March 2009 to check for recent publications of any trials which initially appeared to be unpublished. Duplicate trials were removed. There were no language restrictions.

### Classification of trial completion status

We assigned a completion status of complete, ongoing, temporarily suspended, planned, never started or unclear to unpublished trials. We classified trials as complete if the most recent source reported results and gave no indication that these results were either interim or preliminary, or that patient recruitment or data analysis would continue. If the trial could not be classified as complete after reviewing the most recent source, we searched for the trial using the acronym or intervention on three online databases (Clinical trials.gov http://www.clinicaltrials.gov, The Internet Stroke Center http://www.strokecenter.org and Current Controlled Trials http://www.controlled-trials.com. Trials were classified as complete or ongoing depending on the status described by these databases. If completion status remained unknown, we attempted to contact investigators at least twice. If investigators did not reply by 31^st ^December 2008, the trial completion status was classified as unclear.

### Inclusion criteria

We included all completed randomised controlled trials (RCTs) and controlled clinical trials (CCTs) of pharmacological interventions for acute ischaemic stroke which were unpublished as of March 2009. Trials were labelled as RCTs if they stated that participants or groups of participants were randomly assigned to treatments. We assumed that trials that were described as either double-blind or placebo-controlled or both were randomised. Trials were labelled as CCTs if they stated that participants or groups of participants were allocated prospectively to different interventions using either a quasi-random method, or if no method was described[13]. We defined 'acute stroke' as a stroke assessed within 30 days of symptom onset. We excluded trials which included mixed populations of patients with acute and non-acute stroke unless they reported results for patients with acute stroke separately. Similarly, we excluded trials which included mixed populations of patients with ischaemic and haemorrhagic stroke unless they reported results for patients with ischaemic stroke separately. We excluded trials which did not describe a control group.

### Data extraction

One author (LG) extracted data on trial designs, methods, and results. We recorded sample size, number of centres, type of intervention, comparison groups, method of randomisation, blinding procedures, and allocation concealment for each trial. If details were unclear, trial sources were referred to a second author (PS and/or MB). We classified trials as single or multi-centre according to descriptions provided by investigators. If such descriptions were not available, we noted investigators' institutional affiliations and arbitrarily classified trials with one affiliated hospital or clinic as single centre, and trials with more than one affiliated hospital or clinic as multi-centre, accepting that in rare instances, investigators from multiple hospitals or clinics would participate but only one centre would be responsible for recruiting patients.

We extracted all available data on treatment effects on clinical outcomes (e.g. death, death or dependency and adverse effects) and on non-clinical outcomes (e.g. physiological variables or laboratory measures of uncertain clinical significance). We classified results as qualitative or quantitative and noted whether statistical significance was reported. We extracted data on the most important clinical outcome reported, such as death or functional scales, from the most recent source. In the absence of data on death or dependency, we extracted data on the studies' pre-defined primary outcome. If no primary outcome was defined, we extracted data on the first outcome described in the methods.

We classified the reported effects of interventions as beneficial, harmful, or neutral based on statistical significance, or percentages of participants having a particular outcome if statistical data were not available. We defined trials as beneficial if the intervention was reported to have had a favourable effect compared with the control treatment; as harmful if the treatment effect was adverse; and neutral if no differences were reported between intervention and control groups. Where only quantitative results were available but statistical data were not provided, we classified trials as neutral only if the results were exactly the same for each intervention group. Where results were available for different time points, we recorded only the latest time point. We classified results as 'unable to analyse' if results were not reported separately for each intervention group.

We also extracted data on the number and type of sources reporting a trial, the investigators, country of origin, funding, and date of the latest source. If an investigator appeared to have been involved in multiple completed but unpublished trials, we cross-referenced their institutional affiliation in the sources for the different trials. We contacted investigators if it was still unclear whether they had been involved in multiple trials. We used the institutional affiliation of the first investigator of the latest source to determine the country of origin of each trial. We classified trials as receiving funding from pharmaceutical companies when this was explicitly stated in a source or if any of the investigators were affiliated to a pharmaceutical company.

If any of these data could not be extracted from the most recent source, we checked earlier sources. If data were still not available, they were classified as not reported.

### Calculating the prevalence of unpublished studies

We did not have the resources to fully check the eligibility of each of the published studies. We therefore estimated the proportion that would not meet all our eligibility criteria by assessing in detail a random 20% sample of the published studies. We listed published studies by date of most recent publication, and used random.org [14] to select 20% of studies in each of the following date ranges: 1980-1984, 1985-1989, 1990-1994, 1995-1999, 2000-2004, 2005-2009, pre-1980 and unknown date. The percentage of published studies which otherwise met the inclusion criteria (which we term 'eligible published studies') was calculated and applied to the entire list of published studies. To calculate the point prevalence of unpublished studies, we divided the number of unpublished studies by the sum of the number of unpublished studies and the number of eligible published studies. We used the normal approximation method to calculate 95% confidence intervals (CI) [15].

## Results

### The prevalence of completed but unpublished trials

**Figure 1:**
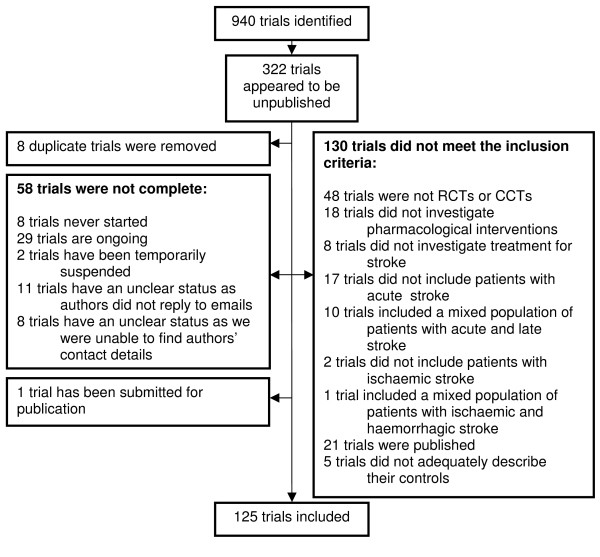
**Trials flowchart**.

The search identified 940 trials of pharmacological interventions for acute ischaemic stroke of which 125 [16-130] (Castillo J: Cooling therapy for acute stroke, unpublished, Chen O: Batroxobin (DF-521) in the treatment of acute cerebral infarction, unpublished, Egberts JFM, Sommer W: Multicentre, randomised, assessor-blind, dose-comparative study of org 10172 (orgaron (r)) in the treatment of acute ischaemic stroke, unpublished, Garcia Tigera J, Avarez LG, Hernandez MO: Treatment with calcium channels blockers (nifedipine) of patients with acute brain infarction, unpublished, Geeganage C, Bath PMW: An assessment of the effects of altering blood pressure on cerebral and systemic haemodynamics in patients with acute ischaemic stroke, unpublished, UCB Pharma: Piracetam vs placebo in the acute treatment of acute ischaemic supratentorial cerebrovascular accidents: pilot study, unpublished, Bath P: Dose escalation study of dobutamine in acute stroke patients, unpublished, Utsumi H: Evaluation of the use of ticlopidine, an antiplatelet agent, in acute cerebral infarction, unpublished) were complete but unpublished by March 23^rd ^2009 and were included in this review (Figure 1). Note that two of the references to the 125 included trials reported two trials each. Our sample of 20% of the published studies identified 21/124 (16.9%) which failed to meet inclusion criteria. Two studies did not test pharmacological interventions, three studies did not involve patients with acute stroke, four did not test interventions for stroke (one study was a secondary prevention trial and three investigated treatment for deep vein thrombosis in patients with stroke), and the remaining 12 studies did not appear to be either CCTs or RCTs. Assuming 16.9% of published studies were ineligible, we estimate that of the 618 published studies, 104 were ineligible published studies and 514 were eligible published studies. The point prevalence of unpublished studies was 19.6% (125/(125+514), 95% CI 16.5-22.6%).

### Available data on design of unpublished trials

**Figure 2:**
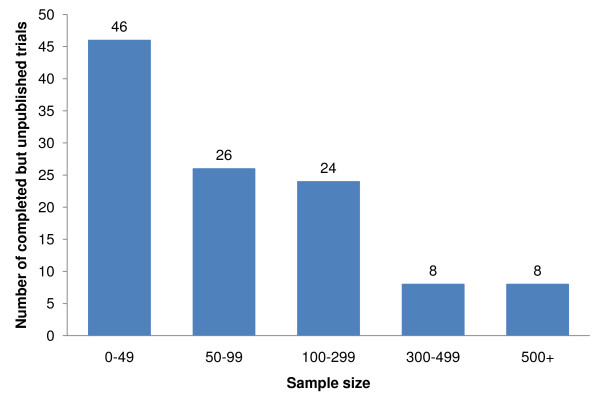
**Actual sample sizes of completed but unpublished trials**.

Of these 125 trials, 112 (89.6%) provided the number of enrolled patients with acute ischaemic stroke (16,058 patients) on whom 89 different pharmacological interventions were tested (Figure 2).

Amongst the 112 trials that provided the number of participants, the mean sample size was 143.4. Two trials included fewer than 10 patients each, and the largest trial included 856 patients (See Additional file 1: Trials which included 100 or more patients). Seventy-two trials included 99 patients or fewer, and 40 trials included 100 patients or more, of which eight trials included over 500 patients. Seventy-three trials reported the number of patients in each treatment group, giving a mean of 60.6 participants per group, with a mode of 2 groups per trial. There were 50 (40.0%) multi-centre trials and 55 (44.0%) single centre trials. We could not determine the number of centres for 20 (16.0%) trials.

Each of the 89 different interventions was investigated by a mean of 1.4 trials (range 1-4, mode 1). Arundic acid, also known as ONO-2506, was investigated by four trials. Seven interventions were investigated by three trials each (cerebrolysin, emoxipin, GM1, insulin, low molecular weight heparin, piclozotan, and piracetam). The 40 trials which included over 100 patients investigated 34 different interventions, with five interventions being tested by two trials each (arundic acid/ONO-2506, eliprodil, GM1, heparin, and piracetam).

Sixty-seven (53.6%) trials were placebo-controlled, of which 23 recruited over 100 patients. The remaining trials used either open controls (n = 29, 23.2%), or the controls received another drug (n = 28, 22.4%). One trial initially stated that controls received placebo and later reported that they received 'conventional treatment' within the only available source.

Details on randomisation, blinding procedures, and allocation concealment methods were often insufficient. Only seven (5.6%) trials - three trials involving over 100 patients - reported the method of randomisation or the allocation to intervention groups. Sixty-eight (54.4%) trials reported their blinding procedures: 10 (8.0%) were single-blind and the remaining 58 (46.4%) were double-blind. Of the 40 trials which recruited over 100 patients, three (7.5%) were single-blind and 21 (52.5%) were double-blind. Just four (3.2%) trials, two of which recruited over 100 patients, reported the method of allocation concealment (See Additional file 1: Trials which included 100 or more patients). Only 29 (23.2%) trials reported the trial phase (mode phase II, range I-III).

### Available data on results of unpublished trials

Of the 125 included trials, 98 (78.4%) trials reported some outcomes. Only 22 (17.6%) trials reported deaths, having recruited 4,251 patients of whom 636 (15.0%) died. Only 6 (4.8%) trials reported the number of patients who either died or became dependent, with a total of 663 dead or dependent patients.

Adverse effects were observed in 40 (32.0%) trials, in 11 (8.8%) trials no adverse effects were noted, and 54 (43.2%) did not provide information on adverse effects. The remaining 20 (16.0%) trials did not provide findings.

We were able to extract data on outcomes reported at the end of the study period for 101 (80.8%) trials. Four (3.2%) trials reported only interim results and 20 (16.0%) trials did not provide any outcome data. Five of the 105 trials which reported outcomes did not provide results according to intervention group, leaving 100 trials with potentially analysable results (Table 1) of which 99 provided information on outcome measures. The overall findings of the European Eliprodil trial were only reported in the medical press without details of the outcomes measured in the trial[131].

**Table 1:** Characteristics of available data in completed but unpublished trials*

	Beneficial (n = 56) n (%)	Harmful (n = 7) n (%)	Neutral (n = 37) n (%)
**Results**			
Final results	54 (96.4)	6 (85.7)	36 (97.3)
Interim results	2 (3.6)	1 (14.3)	1 (2.7)
Clinical outcomes	40 (71.4)	7 (100)	32** (86.5)
Non-clinical outcomes	16 (28.6)	0 (0)	4 (10.8)
**Statistical significance**			
Statistics reported	24 (42.9)	1 (14.3)	7 (18.9)
Statistics not reported	32 (57.1)	6 (85.7)	30 (81.1)
**Nature of results**			
Quantitative	31 (55.4)	7 (100)	13 (35.1)
Qualitative	25 (44.6)	0 (0)	24 (64.9)

*Only 100 trials are included in the table as 20 did not report any results and 5 did not report results separately for each treatment group and were classified as unable to analyse.

** One trial reported results in the medical press, but the outcome measure was not described.

### Available data on other characteristics of unpublished trials

#### Investigators

We noted 551 different investigators as authors of completed but unpublished trials. The mean number of investigators per trial was 5.3 (range 0-18, mode 1). Two trials were investigated by a named group, and the number of investigators for these two trials was classified as 0. Seventy-two different investigators were authors of multiple trials (three were authors of four separate unpublished trials each).

#### Country of origin

**Figure 3:**
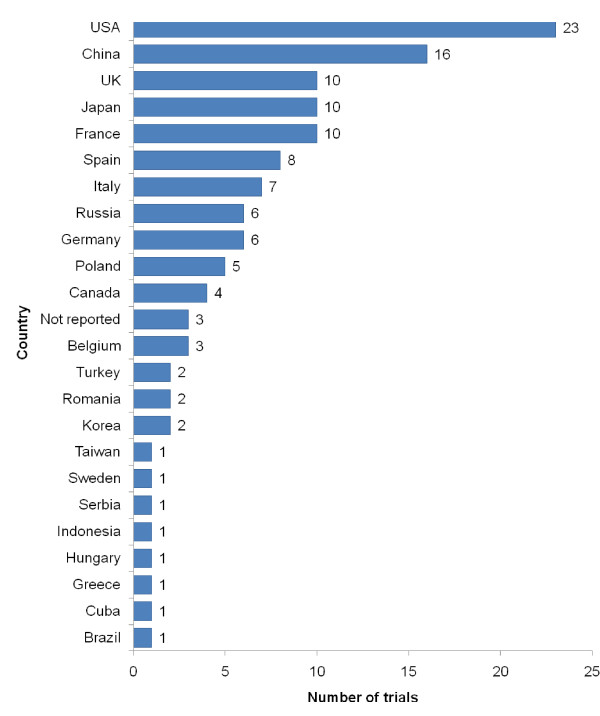
**Country of origin of completed but unpublished trials**. Country of origin was determined from the institutional affiliation of the first investigators listed in the most recent source from each trial.

Completed but unpublished trials were performed by investigators based in 23 different countries (Figure 3).

Only 43 (34.4%) trials reported their source of funding. Thirty-nine trials were funded by pharmaceutical companies and four by other agencies.

#### Sources of information about unpublished trials

Data from unpublished trials was obtained from 238 sources, including 160 (67.2%) abstracts and 78 (32.8%) non-abstract sources such as press releases, letters to editors, protocols, correspondence with investigators, correspondence between investigators and ethics committees, online trials registers, pharmaceutical company reports, and university websites. The mean number of sources per trial was 1.9 (range 1-6, mode 1) with a mean of 1.3 (range 0-5, mode 1) abstracts per trial and a mean of 0.6 (range 0-4 mode 0) non-abstract sources per trial. Fifteen trials were not reported in abstracts. Five trials were identified through online trials registers alone.

#### Dates of most recent report from each unpublished trial

**Figure 4:**
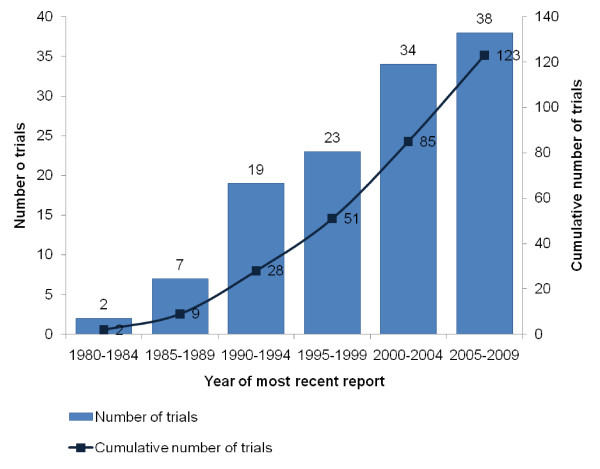
**Year of most recent report from completed but unpublished trials**. Only 123 trials are included in the figure as we were not able to determine the date of the most recent report for two trials.

The number of completed but unpublished trials has increased steadily (Figure 4).

## Discussion

### Key findings

We have demonstrated that an important minority of completed clinical trials are not reported in full, several of which may be large enough to influence clinical practice and the findings of systematic reviews and meta-analyses.

### Prevalence of completed but unpublished trials in other studies

Estimates of completed but unpublished trials in a range of medical fields are much higher than the estimate of this review of acute stroke trials (19.6%). It is difficult to judge if the results of this review of trials in acute stroke are representative of publication practices in other areas of the medical literature (other studies on this question are limited), but we are not aware of any reasons to expect that they would be substantially different. Blumle and colleagues[7] found that 47.6% (49/103) of trials submitted to a German research ethics committee (REC) in 2000 were not published by 2007[7]. Peinemann and colleagues[132] identified 13 trials of negative pressure wound therapy, a type of wound dressing, of which four (30.8%) were unpublished[132]. The difference between estimates may be explained by the small sampling frames used in these studies compared to our review. We used the Cochrane Stroke Group's Specialised Register of Trials, an extremely large collection of stroke trials[11] as the sampling frame. It is the result of a comprehensive search strategy which includes bibliographic databases, trial registry websites, hand-searching of journals, conference proceedings, press releases, correspondence with investigators and information from pharmaceutical companies[12]. There are no language restrictions in the Register which has minimised the risk of selection bias.

Our findings are further strengthened by the system we used to determine the completion status of trials. Previous studies failed to determine completion status of trials so ongoing trials may have been misclassified as unpublished[4,6,9]. Using trial sources, online registers, consultation with an expert and contacting investigators as a last resort, enabled us to determine the completion status of 165 of the 184 trials which met the inclusion criteria.

We identified 40 completed but unpublished trials which had each involved over 100 patients, with eight including over 500 patients each. Large trials are given more weight in meta-analyses, and have greater potential to change clinical practice[133]. The assessment of trial methodological features (e.g. sample size, methods of randomisation, blinding procedures, allocation concealment) is required to decide whether a trial is of sufficient quality for inclusion in a systematic review, meta-analysis, or clinical practice guideline[133,134], but few trials reported sufficient study details to allow methodological quality to be assessed.

### How can authors be encouraged to publish trials more fully? The implications of this review

Initiatives to encourage investigators to disclose unpublished trials have been unsuccessful. Only 165 brief reports of unpublished trials had been submitted to the Medical Editors' Trials Amnesty after one year[135]. A survey of over 40,000 obstetricians revealed just 18 unpublished trials which had been completed at least 2 years previously[136].

RECs, journals and government could help to encourage investigators to publish clinical trials [137-139]. In the UK, it is recommended that RECs require investigators to declare their intent to publish, but it is not yet mandatory (Hugh Davies, unpublished data, 2008). RECs could act at three different stages of clinical trials to encourage publication of the results. Firstly, RECs could require investigators to intend to publish the results[137] when the trial is submitted for ethical approval. Secondly, as RECs currently require investigators to outline a plan of research dissemination [140], they could also request regular updates on the progress of publication after trial completion. Finally, RECs could elicit investigators' reasons for failing to publish results[137] if trials have not reached the public domain within a reasonable time.

Journals have taken steps to encourage investigators to submit reports of trials. Cancer Epidemiology, Biomarkers and Prevention created a section entitled 'Null Results in Brief'[141]. The journal Pediatrics announced that publication of a trial protocol would come with a provisional acceptance of the complete trial report for publication[138]. Journals dedicated to publishing null results now exist, including the Journal of Negative Results in Biomedicine[142]. Established journals could create sections for short reports of trials with null results, and with the development of online publishing, creating space for additional publications has become less of an issue[139].

Prospective registration of clinical trials may help to hold investigators to account for publishing results[139]. Only five trials were identified using online registers alone, suggesting that currently, prospective registration can assist in indentifying unpublished trials, but should not be used as the primary method of locating unpublished trials. Although the CONSORT statement supports prospective trial registration[143], and many journals have become signatories to the statement[144], it is not clear how often journals use prior registration of a trial as a pre-requisite for publication of the results paper. Chalmers suggests that government regulation is also required to improve the reporting of clinical trials[145]. In March 2008, the National Research Ethics Committee, the James Lind Alliance, and the Department of Health announced a joint project called 'Mind the Gap' (Linda Burridge, unpublished data, 2008) with the International Standard Randomised Controlled Trials Number Register and clinicaltrials.gov to improve the completeness of clinical trial registration, and to improve the dissemination of trial results[146]. The project is due to be completed by the end of 2008[146]. Both Italy and America have made comprehensive information on clinical trials publicly accessible[146,147]. Registration of clinical trials investigating drugs and devices subject to approval from the Food and Drug Administration and reporting of basic results online has been a legal requirement in the United States since 2007 [147], but the governments of other countries could follow suit in an international effort to reduce non-publication of clinical trials.

### Limitations

We were unable to determine the status of 19 trials and some may be completed but not published. The status of some trials may have been misclassified if the online trials registers were not up to date. We used the online registers to classify the status of 8 included trials, so any misclassification through using these registers is minimal. Also, the number of centres of some trials may have been misclassified.

We may have overestimated the prevalence of unpublished trials by including trials which were completed within the last three years and trials with very small sample sizes (two trials included fewer than ten patients each). It may be unreasonable to expect such trials to be published. We included all trials completed up to March 2009, and reported point prevalence, as there are now opportunities simultaneously to present trials at conferences and publish them in a journal. The estimates of the proportion of patients who died, were dead or dependent and who suffered adverse effects are of interest, but may not be reliable as the sources available to us rarely reported these data.

## Conclusions

In this review, 19.6% of the clinical trials identified from the register were completed but not published in full. These data represent a substantial body of evidence that is of relevance both to clinical practice in acute stroke and to future research in the field, yet is not fully publicly available. Responsibility for non-publication lies with investigators, but pharmaceutical companies, research ethics committees, journals and governments can all play a part in ensuring that the results of RCTs are made publicly available within a reasonable interval of trial completion.

## Abbreviations

BD: Twice daily; CCT: Controlled clinical trial; FISS-bis: Fraxiparine in ischaemic stroke study; GM1: Monosialotetrahexosylganglioside; IV: Intravenous; LMWH: Low molecular weight heparin; MRECT: Modified randomized exposure controlled trial; MRS: Modified Rankin Scale; NIHSS: National Institutes of Health Stroke Scale; OD: Once daily; PASS II: Piracetam acute stroke study II; PRISTINE: Praxilene in stroke treatment in northern Europe; RANTTAS II: Randomized trial of high dose tirilazad in acute stroke; RCT: Randomised controlled trial; REC: Research ethics committee; RREACT: Rapid response with an astrocyte modulator for the treatment of acute cortical stroke; SC: Subcutaneous; SIST: Sipatrigine in Stroke Trial; TNK-TPA: Tenecteplase - tissue plasminogen activator; tPA: Tissue plasminogen activator; UK: United Kingdom; USA: United States of America.

## Competing interests

The authors declare that they have no competing interests.

## Authors' contributions

LG, MB and PS were responsible for the overall planning and conducting of the systematic review. BT contributed to the literature searches and identification of unpublished evidence. LG screened the search findings, assessed studies for inclusion, extracted and analysed data. LG, MB and PS contributed to interpret results. LG wrote the first draft of the manuscript with additional input from MB and PS. All authors have seen and approved the final version of the manuscript.

## Authors' Information

Lorna Gibson is a medical student at the University of Edinburgh, this work was undertaken as part of her curriculum. Miriam Brazzelli has a Research Training Fellowship in Health Services Research, University of Edinburgh, funded by the Scottish Executive Health Department Chief Scientist Office. Brenda Thomas is the Information Scientist and Trial Search Co-ordinator for the Cochrane Stroke Group. Peter Sandercock is a neurologist with an interest in stroke and is the Co-ordinating Editor of the Cochrane Stroke Group.

## Supplementary Material

Additional file 1 **Trials which included 100 or more patients**. This data file contains data on trial design, sample size, interventions, number of deaths, number of patients dead or dependent, main outcomes and funding sources of trials which included 100 or more patients. Click here for file
